# An assessment of machine learning methods to quantify blood lactate from neutrophils phagocytic activity

**DOI:** 10.1038/s41598-025-90883-7

**Published:** 2025-02-24

**Authors:** Muhammad Nabeel Tahir, Kurt Wagner, Umer Hassan

**Affiliations:** 1 Department of Electrical and Computer Engineering, Rutgers The State University of New Jersey, Piscataway, NJ 08854 USA; 2 Global Health Institute, Rutgers The State University of New Jersey, Piscataway, NJ 08854 USA; 3 Department of Biomedical Engineering, Rutgers The State University of New Jersey, Piscataway, NJ 08854 USA; 4 Present Address: Medtronic, Inc., Minneapolis, Minnesota USA

**Keywords:** Biomedical engineering, Biomarkers

## Abstract

Phagocytosis is a critical component of innate immunity that helps the body defend itself against infection, foreign particles, and cellular debris. Investigating and quantifying phagocytosis can help understand how the immune system identifies foreign particles and how phagocytosis relates to other biomarkers, e.g., cytokines, cell surface receptors, or blood lactate levels. In particular, increased blood lactate levels can be a potential biomarker to study diseases, e.g., septic shock. Establishing a relationship between phagocytosis and lactate levels can serve as an effective tool to monitor the immune response and may help stratify patients. In this study, we use phagocytosis activity data to classify the patients into two groups of blood lactate levels (High and Low) with machine learning models. The neutrophils extracted from the whole blood samples of 19 patients were used to collect data on phagocytosis, where the neutrophils were allowed to internalize IgG coated fluorescent bioparticles. The data collection process involved collecting whole blood samples, neutrophil isolation, adding fluorescent beads, incubating, and imaging the sample using a fluorescence microscope. The phagocytosis assay images were used to generate a numerical dataset by manually counting the number of particles engulfed by each cell. The study first presents an improved understanding by employing hierarchical clustering and heatmaps to generate the graphical representation of phagocytosis data. By comparing the results of heat maps and clustering techniques, it can be observed that the phagocytosis activity data can be used to differentiate blood lactate levels in two groups (control and high-risk). Later, three machine learning models (Decision Tree, k-nearest Neighbor, and Naïve Bayes) were trained on the original and pruned datasets after the outliers were removed. The AI models classified the data into high-risk and low-risk groups of blood lactate levels. A maximum classification accuracy of 78% and an area under the curve of 0.78 was achieved using the trained models.

## Introduction

Phagocytosis is an essential cellular process of the immune system. During this process, certain white blood cells (neutrophils, macrophages, monocytes, etc.) called phagocytes engulf the attacking microorganisms, e.g., bacteria, and digest them^1^. Multiple data analysis techniques and methods have been developed to quantify the phagocytic activity and draw inferences regarding a particular disease’s state and progression, e.g., Sepsis, HIV, TB, etc. Studies^2–5^ have employed deep learning techniques to quantify phagocytosis and its relation to the disease under consideration. Due to the complexity and requirement of powerful computational resources of deep learning techniques, standard statistical methods have also been employed to analytically infer complex patterns and draw conclusions from the phagocytosis data discussed in studies^6,7^. Similarly, computationally inexpensive machine learning methods have been used to detect biomarkers or diagnose diseases under consideration, e.g., k-nearest neighbor, decision trees, or naïve Bayes classifier, etc^8,9^. These studies utilize fluorescence imaging or flow cytometry to collect the data and try to determine the effect of phagocytosis or other related biomarkers on the immune system^10,11^. Phagocytosis has also been used to study the potential biomarkers associated with the progression of different diseases, e.g., sepsis, cancer, or COVID-19^12,13^. A relationship between phagocytosis by neutrophils and blood lactate levels has attracted significant interest in immunology and metabolic research. Lactate is a byproduct of anaerobic metabolism, a process by which cells generate energy. During an inflammatory response to an infection, e.g., sepsis, the activated neutrophils release lactate in the blood, increasing the blood lactate levels^14–16^. These lactate levels can act as a signal to other cells to increase their phagocytic activity. Blood lactate levels have long been identified as the potential tissue hypoxia and cellular stress biomarker. A substantial amount of research has been conducted to study lactate levels as a potential biomarker in detecting and diagnosing various diseases. An increase in blood lactate levels is associated with increased mortality in patients with sepsis^17–22^. Sepsis is a severe life-threatening disease and occurs when the immune system starts damaging healthy tissues and organs in response to an infection. During sepsis, the neutrophils are activated and release large amounts of lactate in the blood during phagocytic activity, thus making lactate levels a biomarker for sepsis. A decreased oxygen supply to the organs and tissues worsens the inflammatory response. Lactate production has also been studied as a biomarker in the mobilization and recruitment of inflammatory cells and neutrophils^16,23^. The relationship between microglial phagocytosis and lactate levels has also been explored in the study of Alzheimer’s disease^24^. Moreover, lactate levels have been employed as a potential biomarker in developing a therapy for prostate cancer^25^. These studies report several mechanisms that try to explain the impact of lactate levels on inflammatory cells or neutrophil phagocytosis. The studies show that lactate regulates immune cell function by affecting intracellular signaling pathways. Furthermore, lactate can influence the environment surrounding neutrophils, including pH regulation and the production of reactive oxygen species, which is crucial for phagocytosis.

Exploring the relationship between blood lactate levels and phagocytosis by neutrophils can unveil a complex and intricate interplay between metabolism and the immune system. It may also provide insights into how increasing or decreasing lactate levels affect the functioning of immune cells, especially neutrophils, and the overall balance of the immune system. Moreover, exploring this relationship may have implications for various pathological conditions associated with altered lactate metabolism, including sepsis, cancer, and inflammatory disorders. In this paper, we study the phagocytic activity of human neutrophils by using fluorescently tagged beads mimicking pathogens. We explore analytical inference techniques to understand better phagocytosis and its association with increased blood lactate levels. Machine learning models have been developed to investigate the relationship between phagocytosis and lactate levels. The developed algorithms classify the data into two groups (low and high-risk) of blood lactate levels. The diagnostic abilities of these classifiers are compared and evaluated in relation to our previous study^26^.

**Fig. 1:**
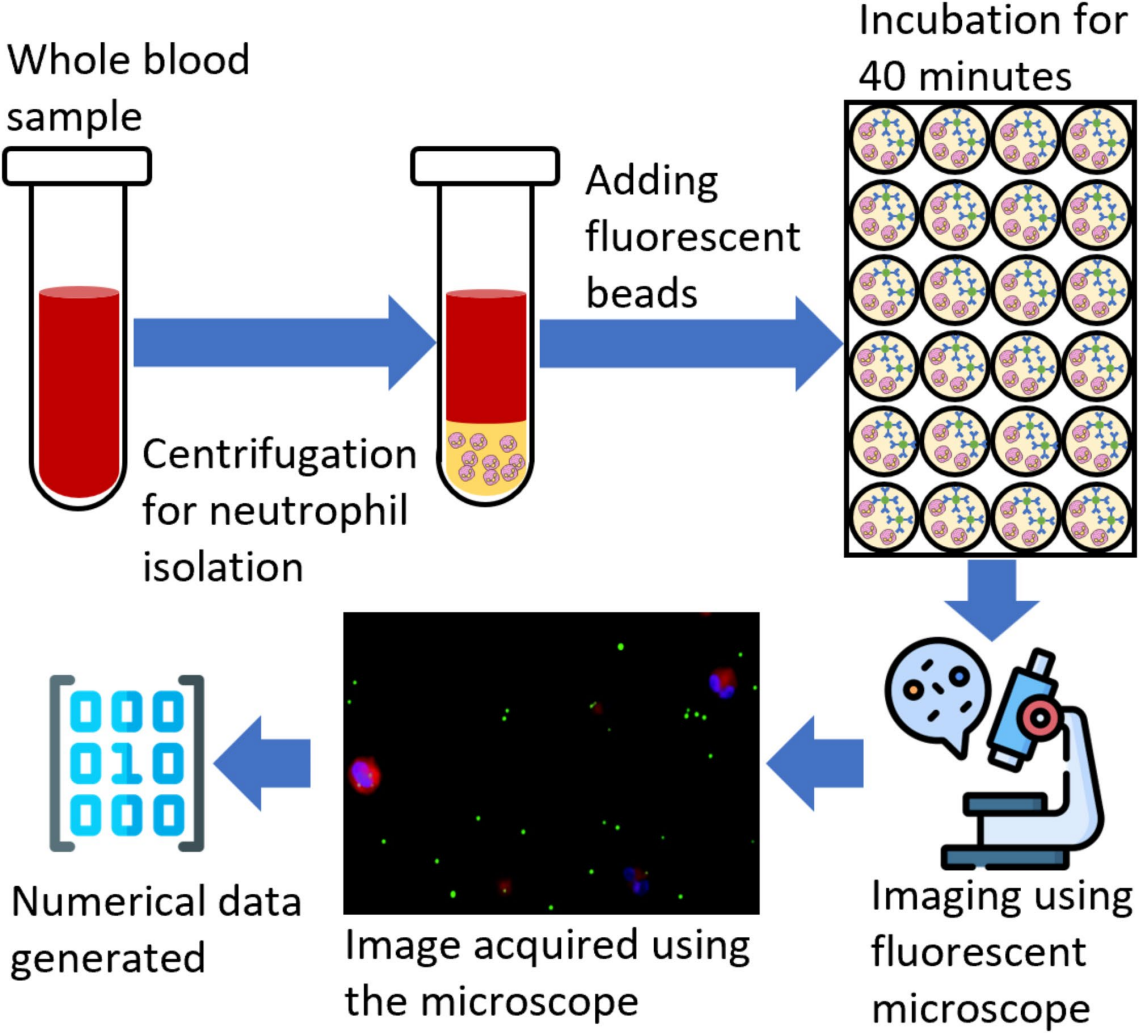
The flow chart of the experimental procedure followed to collect the phagocytosis data of neutrophils.

## Materials and methods

### Experimental setup and data collection

This study follows the experimental setup and data collection protocol discussed in the previous study^26^. Blood samples were collected from the Robert Wood Johnson Hospital under IRB (IRB application # Pro2018002356) for 19 patients to study the human neutrophils’ phagocytic activity and their relation with blood lactate levels. Out of 19 patients, eight were clinically diagnosed with lactate levels lower than two mmolL^− 1^, while the rest had higher than 2 mmolL^− 1^. The data collection and preprocessing were completed in three steps. During the first step, the neutrophils were isolated from the whole blood sample by following a standard cell isolation protocol. The whole blood mixed with 1x phosphate-buffered saline (PBS) was layered over a density gradient in centrifuge tubes. After centrifugation, the polymorphonuclear leukocytes (PMNL), erythrocytes, and red blood cells formed a dense layer at the bottom of the micro-tubes. The top layers above the erythrocytes were removed, and the red blood cells were lysed. The remaining leukocytes were pelleted and washed with PBS. To improve the purity, erythrocytes were lysed, and the isolated PMNL were suspended in the RPMI 1640 medium and counted. As observed, the isolation process resulted in high purity and the capture efficiency of neutrophils up to 95%.

In the second step, the neutrophils were added to a 24-well plate at a concentration of 6 × 10^5^ cells per well. Each patient’s blood sample was tested in triplicate to improve the test accuracy. Fluorescent beads tagged with human IgG antibodies that resemble common bacteria in size were added to the wells at a ratio of 1:25. A cell membrane dye was added to visualize the cell surface. The solution was incubated for 40 min at 37^◦^C to facilitate phagocytosis. After incubation, the remaining beads were removed and washed with chilled PBS, and a nucleic acid stain was added. The solution was kept on ice for 10 min and washed with PBS. The media was imaged using a fluorescent microscope, and four images were collected for bright field, blue nucleic acid stain, orange cytoplasmic stain, and green antibody stain. These images were combined by adding, and a single image was generated.

Finally, an open-source image processing software, ImageJ^27^ was used to quantify the phagocytic activity. A total of 15 images were analyzed for each experiment, each well contributing five images. Initially, the regions fluorescing orange cytoplasmic membrane dye and blue nucleic stain dye were identified. These regions correspond to the number of cells and beads inside the areas of interest, which were counted as the internalized particles. The total number of internalized particles was also calculated for each cell. Following this protocol, a numerical dataset was generated for each patient. The dataset consists of ten features and 19 examples of phagocytosis activity^26^. Features include the “average and standard deviation” of beads internalized by the cells and the counts of cells with “zero to seven” beads internalized, resulting in 10 features. The dataset was then normalized by subtracting the minimum feature value and dividing it by the difference between maximum and minimum feature values. Finally, the dataset was split into two groups: a control group (low lactate levels) and a high-risk group (high lactate levels). The resulting group variable was used as the target variable for binary classification. Figure 1 shows the flow chart of the experimental data collection and processing procedure.

### Outlier detection and removal with isolation forest

As stated in the previous study^26^ the machine learning algorithms performed poorly when trained on the collected dataset. One of the primary reasons for the poor performance of the algorithms can be attributed to anomalies in the dataset. To improve the detection and classification ability of the machine learning algorithms an outlier detection and removal algorithm based on the ensemble of isolation trees^28^ has been employed. An anomaly score is calculated for each observation based on the average path lengths over all the isolation trees, as shown in Eq. 1.

1 $$\:s\left(x\right)=\:{2}^{\frac{E\left|h\right(x\left)\right|}{c\left(n\right)}}$$

Where *s*(*x*) is the anomaly score of an observation *x*, *h*(*x*) is the path length in a tree, *E*|*h*(*x*)| is the average path length over all isolation trees, and *c*(*n*) is the average path length of unsuccessful searches in a binary tree of n observations. The contamination factor 0.05 was used as it assumes that approximately 5% of the data is corrupted or contains outliers. Also, the size of the original dataset was small. A conservative approach was used as a higher contamination factor, which might consider normal points to be anomalies and reduce the size of the dataset significantly. Moreover, the chosen value aligns with typical anomaly expectations in real-world scenarios. Each subgroup in the collected dataset was trained using this technique, and the anomalies were removed. Finally, the pruned data groups were concatenated and used in the training and evaluation of an ensemble of bagged trees, an ensemble of k-NN, and Naïve Bayes classification algorithms.

### Ensemble of bagged decision trees

In this study, a bagged decision tree and random forest-based ensemble learning technique have been employed. A total of 30 bootstrap replicas were generated for the training data with the replacement of the selected sample. A decision tree was trained on each bootstrapped dataset with a random forest technique that allowed each learner to choose the predictors randomly. The classifier learner app in MATLAB was used to generate the code for bagged decision trees with ensemble learning.

### Ensemble k-nearest neighbor

K-nearest neighbor classifiers with random subspace ensembles have been employed to classify the data into control and high-risk groups. The k-NN is a non-parametric supervised learning algorithm, and the Euclidean distance metric was used to calculate the distance between neighbors. The random subspace ensemble technique in this study selects a random set of features from the training dataset and trains a k-NN model. The k-NN model was trained with k being set to 5 neighbors. Since the dataset size is small, using too few neighbors could lead to prediction instability. At the same time, a bigger value of k can ignore the influence of local patterns in the dataset. Selecting k = 5 provided a balance between ensuring that the model can learn local patterns while avoiding overfitting. The method predicts the final score of the class by taking the average of all the trained classifiers, with the class having the highest average score.

### Naïve bayes classifier

The Naïve Bayes classifier is a statistical machine learning algorithm that leverages the Bayes theorem and makes predictions about the class of the observations based on the conditional probabilities of features given the class labels. The algorithm first estimates the likelihood of the features given a class label for the entire dataset. Then, based on the likelihood and priors, the occurrence of the class variable is estimated based on the observation. In this study, a Naïve Bayes classifier has been trained on the collected data and kernel normalization on the input feature vectors. The kernel normalization assumes a probability distribution on the input feature and normalizes the feature vector. Normal distribution was assumed to fit each feature vector, and the corresponding normalization was applied. A normality test was performed on 8 features, where the eighth feature provides information regarding the 5 or more number particles engulfed. Since the last two features contain spare data, they have been added to the 8th feature. The Kolmogorov-Smirnov method was used, and the p-value was acquired. A significance level of 0.05 was used, and all the features reported a p-value > 0.25, indicating that the features follow a normal distribution.

### Clustergram

A clustergram combines heatmaps and dendrograms to visualize the similarities or dissimilarities between the variables in the dataset. Each value/color in the cell corresponds to the normalized value of that feature for a particular sample. Dark red indicates the high value, white indicates the average value, and dark blue indicates the lowest value. A hierarchical clustering technique groups the items together based on distance or similarity indices. This study uses the correlation distance as the dissimilarity metric. The correlation distance is calculated as shown in Eq. 2, and the similarity/dissimilarity matrix is generated.

2 $$\:{d}_{c}=1-{r}_{xy}$$

Here, *d*_*c*_ is the correlation distance, while $\:{r}_{xy}$Is the sample correlation and is calculated as:

3 $$\:{r}_{xy}=\:\frac{{(x-\stackrel{-}{x})(y-\stackrel{-}{y})}^{{\prime\:}}}{\sqrt{{(x-\stackrel{-}{x})(x-\stackrel{-}{x})}^{{\prime\:}}}\sqrt{{(y-\stackrel{-}{y})(y-\stackrel{-}{y})}^{{\prime\:}}}}$$

The correlation distance quantifies the dissimilarity between two variables or datasets. The higher values of correlation distance suggest that the variables are dissimilar, as the correlation coefficient between the variables will be lower. The lower values of the correlation distance are related to the higher similarity between the variables, as the correlation coefficient will be higher.

### Agglomerative hierarchical clustering

Agglomerative clustering is a type of hierarchical clustering technique in unsupervised machine learning. The algorithm follows a bottom-up approach where it considers each data point as the cluster or singleton cluster, calculates the distance between the clusters, and merges the clusters that are closer to each other in distance. This study employs the Euclidean distance as the distance metric and aggregates the clusters based on the average distance method. First, the distance between each point in the two clusters is calculated using the distance metric. Then, the final distance between clusters is calculated using the distance method, e.g., Average, Centroid, Ward, etc. The clusters are merged based on the threshold set on the average distance. The process is repeated until all the clusters are merged into a single cluster. Starting from the bottom with singleton clusters, the algorithm generates a tree-like structure at each iteration until convergence is reached with the parent node or cluster. Each level of the created dendrogram/tree provides the number of clusters that can be formed based on the distance or similarity index between the variables. Once a dendrogram is generated, it can be used in machine learning or statistical analysis to draw conclusions from the results and analyze the dataset.

### Training procedure and environment

The dataset prepared using the procedure discussed in the experimental and data collection section was then used by the outlier detection algorithm, which resulted in a new dataset. To make fair comparisons between the performance of selected algorithms and the effects of data pruning, all the models were trained on both original and pruned data. As the number of training examples is low and models are susceptible to overfitting, a 5-fold cross-validation technique has been employed to estimate the robustness of all selected machine learning models and reduce the chances of overfitting. Moreover, an ensemble learning technique was used to optimize the performance metrics. A hundred different versions of the training data were generated by randomly shuffling the data. All the models were trained on the 100 generated datasets, resulting in 100 different trained models for each type of machine-learning technique. The accuracies of these models were used to estimate the overall accuracy of the classification. All the models were trained in a system (Core i9, 10th Gen, 32GB RAM) with MATLAB as the development environment.

**Fig. 2:**
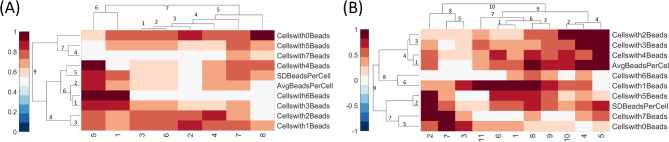
The clustergram plots were generated using heatmaps and dendrograms. (A) The clustergram of the Control group. (B) The clustergram of the High-risk group.

## Results and discussion

### Comparison of the control and high-risk clustergrams

The clustergram can provide valuable insights into the differences between the datasets of patients with high risk or low risk based on the features corresponding to the phagocytosis activity. The clustergrams using the technique discussed in Materials & methods section were generated for each group in the collected dataset. Figure 2 shows the clustergram of samples in the control and high-risk group generated against the ten feature variables in the dataset. The columns of the clustergram represent the samples in the control group Fig. 2 (A) and high-risk group Fig. 2 (B), while the rows represent the feature variables. The figure also shows two dendrograms at the top and on the left side. The top dendrogram creates clusters of samples based on the similarities in the feature variables. Meanwhile, the left dendrogram groups the feature variables based on the data distribution in each sample. A distance metric is calculated for each feature (row) or sample (column), and points with the least distance are grouped together. Using clustergrams to generate heatmaps and dendrograms for control and high-risk data has enabled us to understand the relationship between the samples and corresponding feature vectors. Although the clustergrams have divided the data in both groups into multiple sub-clusters, the clusters follow the same data distribution when merged. The data used to generate cluster-grams is normalized between 0 and 1, indicating that in a sample, the number of beads are engulfed by cells. For example, if a sample contains a high value, i.e., >0.9 in a particular cell, e.g., Cellswith2Beads, it shows that many cells have engulfed two beads. The cells in the heatmap of the control group clustergram show significantly lighter shades of red (0-0.5) compared to the high-risk control group (0.5-1), suggesting that the control group had lower phagocytic activity as the number of particles engulfed was lower. Even though the dataset is small, the results provide a preliminary indication about the phagocytic activities of both the control and high-risk groups, which should be further evaluated for more concrete results. All the samples in the control and high-risk groups show darker shades of the feature ‘Cellswith0Beads (> 0.8)’, indicating that many cells are not involved in phagocytosis even if patients have high or low-risk blood lactate levels. Comparing the features indicating where cells have engulfed more 1, 2, or 3 beads for both groups, it can be observed that the high-risk group contains darker shades of red (> 0.85) than the control group, suggesting higher phagocytic activity. Similar observations can also be made for the other set of features. The left dendrogram of the control group contains a shorter branch of the cluster (4,7), which indicates that the features “Cellswith0Beads”, “Cellswith7Beads”, and “Cellswith5Beads” have more similarities. On the other hand, the left dendrograms of the high-risk group contain more distributed branches, indicating the independence of the features. An important observation that can be made from the top dendrogram of the control group clustergram is that samples 1 and 5 can also be considered anomalies or at borderline risk of high blood lactate levels. The rest of the samples in the control group show similar values for the features, and that’s why they have been grouped into a bigger cluster 5. However, the top dendrogram of the high-risk group contains more uniformly distributed clusters, which suggests that the high-risk group has more variance in sample values. Moreover, a statistical power analysis was performed to estimate the minimum number of samples required to differentiate between control and high-risk groups using G*power software^29^. First, a generalized linear model was fitted on the data, and McFadden’s $\:{R}^{2}$ correlation coefficient was estimated as $\:{R}_{McFadden}^{2}=1-\frac{{L}_{a}}{{L}_{0}}=0.7263$. Where $\:{L}_{a}$is the alternate hypothesis indicating the log-likelihood of the fitted model with predictors and $\:{L}_{0}$ is null hypothesis indicating the log-likelihood of the model without predictors (intercept only). We used the estimated $\:{R}^{2}$and calculated the effect size as $\:{f}^{2}=\frac{{R}^{2}}{{1-R}^{2}}=2.65$ and performed an F-test with linear multiple regression fit with significance level of 0.05 and power of 0.8. The test resulted in an estimated sample size required to be 18 which is within the limits of actual sample size 19 used in this study.

### Machine learning model comparisons

This study’s selected machine learning models were trained on both the original data and processed data after removing the outliers. The pruning methods were able to remove at most two erroneous readings from the original dataset, which resulted in the actual size of the data being seventeen samples. The 100 trained versions of each machine learning technique classify the data into control and high-risk groups and have different convergences. As a result, the trained models have different cross-validation accuracies. To further evaluate the performance of the trained models’ the receiver operating characteristic curve (ROC) of each model was generated. The ROC curves and the validation accuracies of 100 trained models were averaged for ensemble DT, ensemble k-NN, and NB algorithms. The averaged values of these performance measuring parameters depict the overall convergence of the selected machine-learning models. A majority vote from the 100 trained machine-learning models decides the class of the test observation. The reason for selecting these models is precisely the small dataset. As the dataset is small, it’s susceptible to overfitting if larger and more complex models are selected. Therefore, ensemble-based simpler networks were selected. Moreover, shuffling the dataset and training 100 models ensures that the machine learning technique generalizes the dataset and does not either overfit or underfit the data. The hyperparameter of 100 was empirically selected to better quantify the percentage of the models with classification accuracy above an average limit.

**Fig. 3:**
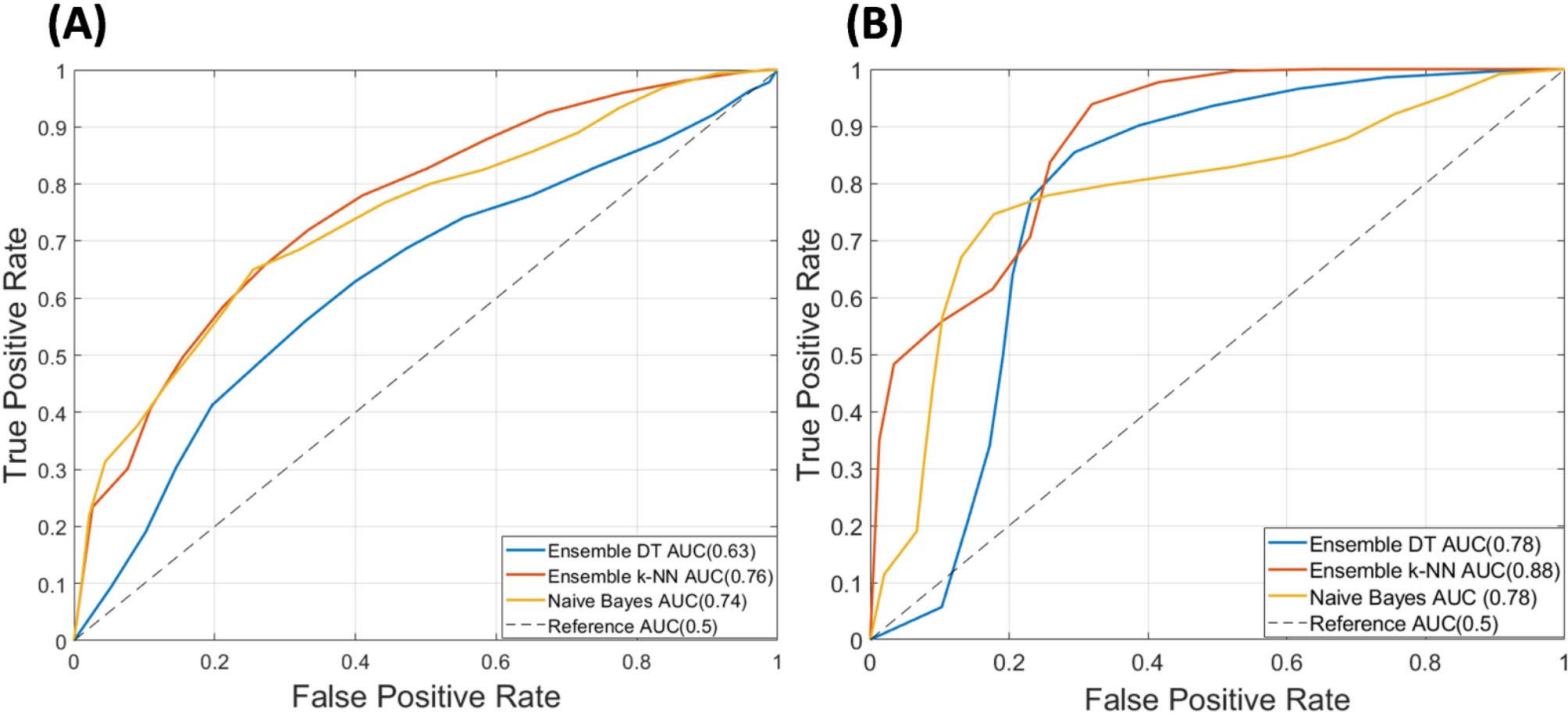
Receiver operating curves of trained machine learning algorithms. (A) The average ROC curves of selected machine learning models on the original dataset. (B) The average ROC curves of selected machine learning models on the pruned dataset.

**Fig. 4:**
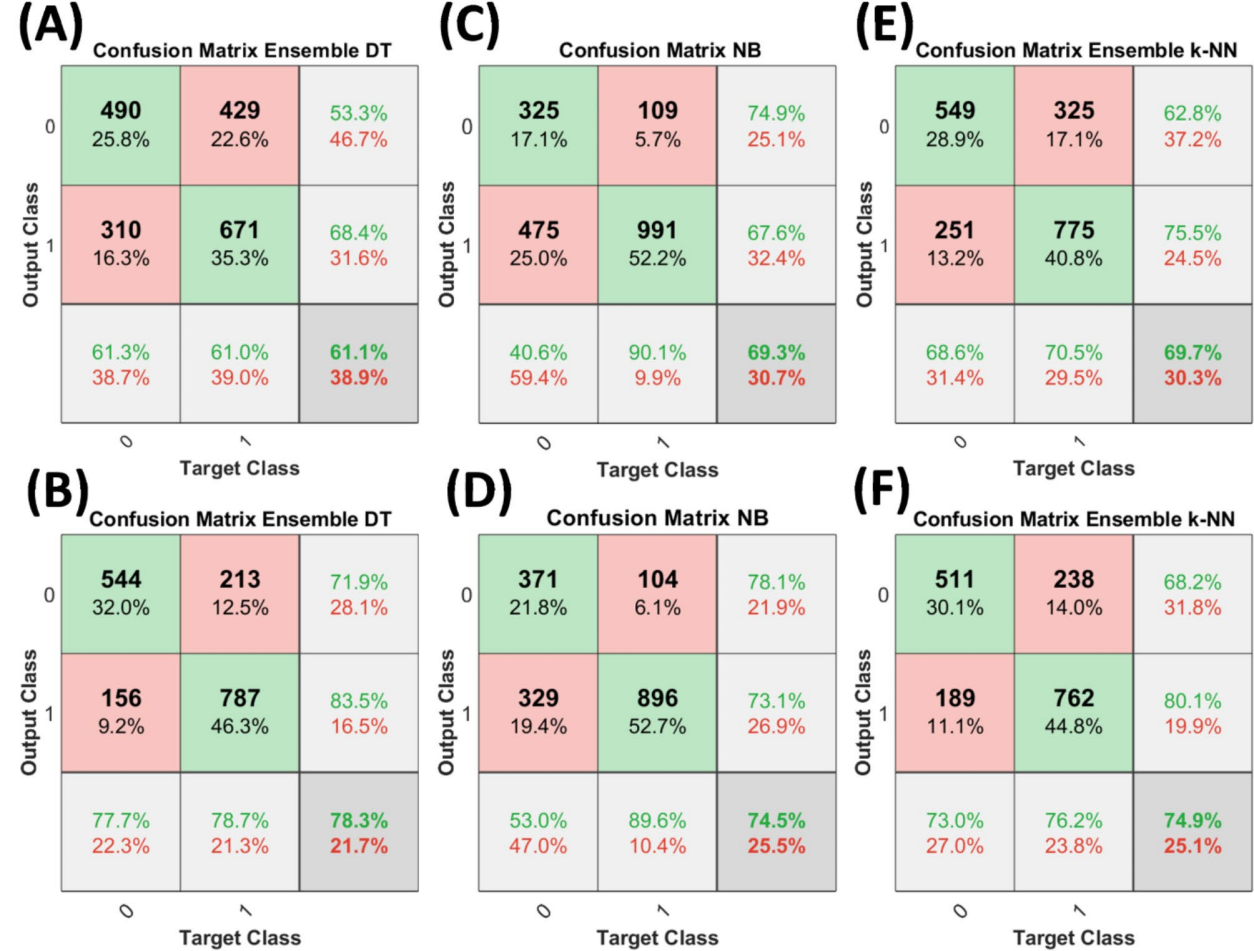
Confusion matrices of training results of selected machine learning models. (A, C, E) Confusion matrices of training results on original data. (B, D, F) Confusion matrices of training results on pruned data models on the pruned dataset.

When trained on 100 versions of original and pruned datasets, the ensemble bagged decision trees generated the average ROC curves, as shown in Fig. 3. On average the ensemble bagged decision tree performed significantly better on pruned data as compared to the original data. An average validation accuracy of 78.3% and an average AUC of 0.782 was achieved with 100 trained models of ensemble bagged decision trees on pruned data. The models performed poorly on the original data, achieving an average validation accuracy of 61.1% and an average AUC of 0.63, as shown in Fig. 3(A). The lower values of AUC show that the networks did not provide accurate discrimination between control and high-risk groups. Figure 4(A) and (B) show the confusion matrices of ensemble bagged decision trees for original and pruned data, respectively, where class “0” is the control group and class “1” is the high-risk group. However, models trained on pruned data performed significantly better. The false positive rate and the false negative rate are higher, as is clear in Fig. 4(B). One possible explanation for such higher false rates is the lower number of training examples in the dataset and the higher number of feature vectors. The overfitting effect in this case is not significant because the reported results are averaged over 100 trained models, and if there had been overfitting, the models would have performed worse. Similarly, the average validation accuracies and average ROC curves were generated for 100 ensemble k-NN and NB classifiers, as shown in Fig. 3 on both original and pruned datasets. The ensemble k-NN performed second best in terms of average classification accuracy and average AUC and achieved values of 74.9% and 0.88 on pruned data and 69.7% and 0.76, respectively. The Naïve Bayes performed third in terms of average classification accuracy and average AUC and achieved 74.5% and 0.78 values on pruned data and 69.3% and 0.74, respectively. It is clear that both ensemble k-NN and NB have almost the same performance on original and pruned data but have higher AUCs than ensemble DT. The higher AUCs in both machine learning models are due to fewer training examples and a class imbalance in the high-risk and control groups. Since the high-risk group contains more training examples than the control group, the learning of ensemble k-NN and NB is skewed towards the high-risk group, and we see fewer false positives and more true negatives in the confusion matrices, as shown in Fig. 4(C-F). This increases the true positive rate, and as a result, a higher AUC is achieved, but the classification accuracies remain lower since the control group has more classification errors.

Overall the ensemble bagged decision tree has higher accuracy when trained on the pruned dataset as compared to the other two machine learning models. At the same time, the same model has the lowest accuracy and AUC on the original dataset as compared to the other two machine learning models. Moreover, the ensemble-bagged decision tree outperformed the machine learning models trained in the previous study^26^ and showed significant improvements in classification accuracy and AUC.

Moreover, the results of the current exploratory study encourage the use of machine learning methods in differentiating patients with low or high risks of sepsis, as the ML models have been employed in the literature for biomarker prediction and disease diagnosis. Although the results discussed here are promising yet there is a significant need for improvement before such a system is deployed in clinical settings. The major limiting factor in the current study is the unavailability of the complete patient data, i.e., sex, age, disease, vitals, and diagnosis. A smaller set of examples can provide encouraging results, but in order to create a generic model, a wide range of samples should be collected and analyzed. Therefore, we aim to extend the study with an updated IRB to collect the patient information and clinical parameters to quantify phagocytosis activity and then develop an improved ML model to distinguish between two groups of interest discussed in the study.

## Conclusion

A numerical transformation of imaged phagocytic activity of neutrophils has been used as a dataset to train three machine learning models: ensemble bagged decision tree, ensemble k-NN, and Naïve Bayes classifiers. The classifiers aim to differentiate between the groups of patients with low or high levels of lactate utilizing the phagocytosis activity data. The cluster-grams generated for each group in the data helped us understand the properties of the individual sub-clusters in the main groups qualitatively and what makes them unique. Moreover, the one-to-one comparison between sub-clusters of control and high-risk groups suggests that both groups have a different set of properties and can be used to train an AI model. An isolation forest-based outlier detection and removal method first removes the faulty observations, and the pruned data is then used to train models. To accurately evaluate the performance and diagnostic ability of the classifiers, 100 random versions of the dataset were created, and 100 models were trained for each category of classifiers. The resulting validation accuracies and receiver operating curves were averaged of all the 100 trained networks, and a unified accuracy and ROC curve were reported. An accuracy of 78.3% was achieved with the ensemble bagged decision tree algorithm outperforming the ensemble k-NN and Naïve Bayes classifiers. With an area under the curve of 0.78, it can be inferred that the trained ensemble bagged decision tree model can be used to classify the phagocytosis data into two groups of lactate levels. In future work, generating more data can improve the classification accuracy and diagnostic ability of the machine learning models so that they can be used as clinical tools. We also aim to explore image segmentation and DL-based feature extraction techniques to extract the number of features or particles engulfed by the cell and use the data to train ML models for prediction. We also aim to utilize the patient information in the machine learning model acquired under the future IRB for improved classification of patients with or without a high risk of sepsis.

## Data Availability

The datasets used and analysed during the current study are available from the corresponding author on reasonable request and based on the policies of funding agencies and Rutgers, The State University of New Jersey.
